# *Theosbaena loko* sp. n. a new stygobiotic microshrimp (Thermosbaenacea: Halosbaenidae) from southern Thailand

**DOI:** 10.3897/BDJ.8.e59528

**Published:** 2020-11-10

**Authors:** Sopark Jantarit, Rueangrit Promdam, Koraon Wongkamhaeng

**Affiliations:** 1 Excellence Center for Biodiversity of Peninsular Thailand, Faculty of Science, Prince of Songkla University, Songkhla, Thailand Excellence Center for Biodiversity of Peninsular Thailand, Faculty of Science, Prince of Songkla University Songkhla Thailand; 2 Princess Maha Chakri Sirindhorn Natural History Museum, Prince of Songkla University, Songkhla, Thailand Princess Maha Chakri Sirindhorn Natural History Museum, Prince of Songkla University Songkhla Thailand; 3 Department of Zoology, Faculty of Science, Kasetsart University, Bangkok, Thailand Department of Zoology, Faculty of Science, Kasetsart University Bangkok Thailand

**Keywords:** new species, peninsular Thailand, subterranean habitats, taxonomy

## Abstract

**Background:**

Thermosbaenaceans are subterranean crustaceans, widespread and occur in freshwater, oligohaline or anchialine caves or thermal springs. Currently, four families, seven genera,and 45 species are recognised worldwide. During our studies of the isolated karst, Tham Loko (Loko Cave) in Khao Chiason District, Phatthalung Province, we found an undescribed thermosbanacean species in the genus *Theosbaena*. *Theosbaena* is the only genus reported from freshwater in the Oriental Region. Previously, there were only two known species, *Theosbaena cambodjiana* Cals & Boutin, 1985 from Kampot Province, southern Cambodia and Khon Kaen, Thailand and *T. kiatwongchai* Rogers & Sanoamuang, 2016 discovered in a cave of Takhli District, Nakhon Sawan, central Thailand. Our new species is the third species recorded in the Oriental Region.

**New information:**

*Theosbaena loko* sp. n. differs from its congeners by having a telson 1.8x longer than its breadth, maxilla 1 palp distal segment 4x longer than the proximal palpomere and the maxillopodal exopod twice as long as its basal width. This microshrimp is the third described species of the genus. A key to the species is given and suggestions for the conservation status of the new species are discussed.

## Introduction

Thermosbaenaceans are generally recognised by their small size (< 5 mm), roughly cylindrical body, short carapace, biramous pereopods, blindness, lack of pigment and the female broods her eggs (Jaume 2008, Sket 2019, Wagner 1994). Thermosbaenaceans are widespread and occur in a variety of habitats. Most of them are oligohaline or anchialine. Some have adapted to freshwater or thermal springs or cold waters (Sket 2019; Wagner 1994). Currently, four families, seven genera and 45 species are recognised worldwide, namely *Halosbaena* (five species), *Limnosbaena* (two species), *Monodella* (one species), *Tethysbaena* (31 species), *Theosbaena* (two species), *Thermosbaena* (one species) and *Tulumella* (three species) (Boyko et al. 2008).

**Figure 1.**
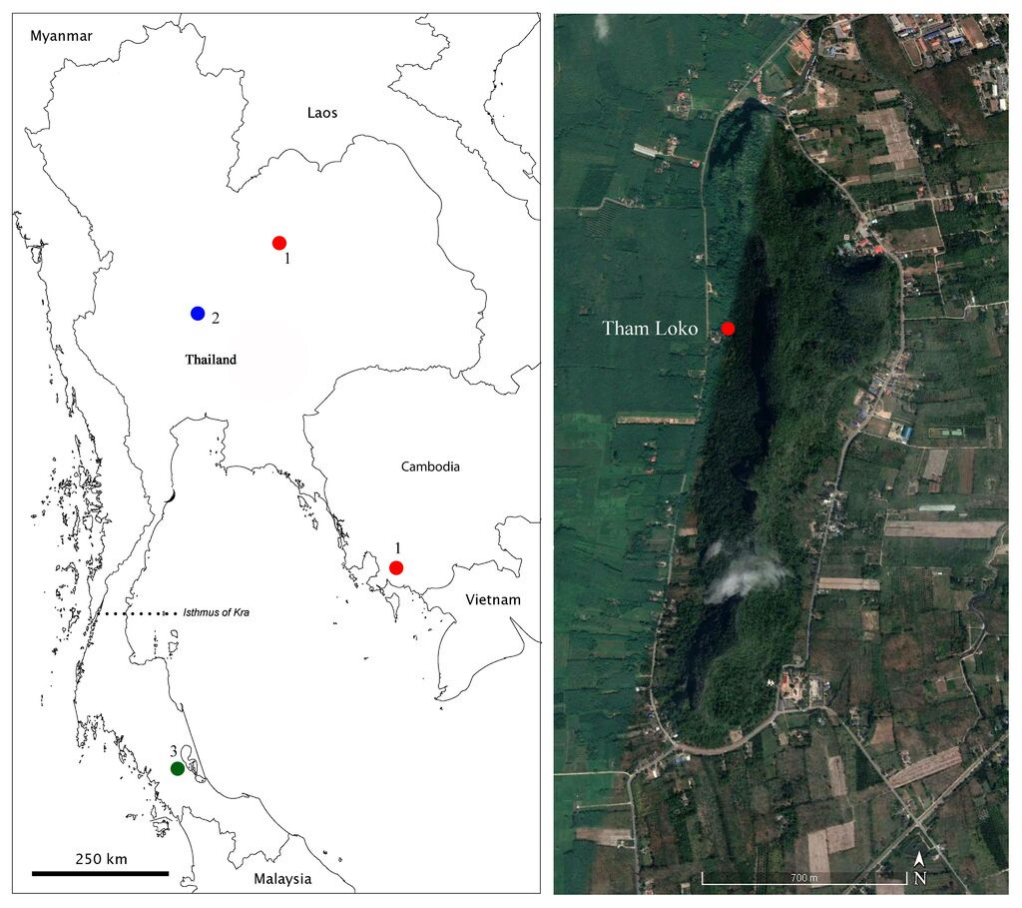
Distribution of *Theosbaena* in the Oriental Region. (Left) *T. cambodjiana* in red circle, *T. kiatwongchai* in blue circle and *T. loko* sp. n. in green circle. (Right) Khao Chaison isolated karst system extracted from Google Earth.

In the Oriental Region, only *Theosbaena* has been recorded in freshwater (Boutin and Magniez 1985). The species in this genus are highly endemic and have a narrow distribution range. All are found in limestone caves with only two known species so far. *Theosbaena cambodjiana* Cals & Boutin, 1985 is reported from the caves of Kampot Province, southern Cambodia and from a cave in Khon Kaen Province, north-eastern Thailand (Rogers and Sanoamuang 2016). The second species is *T. kiatwongchai* Rogers & Sanoamuang, 2016, discovered in a cave of Takhli District, Nakhon Sawan Province, central Thailand (Fig. 1).

Here, we present a third species of the genus *Theosbaena*. The species was discovered in a limestone cave in Phatthalung Province, southern Thailand. We provide a key to the species and discuss its conservation status.

## Materials and methods

**Figure 2.**
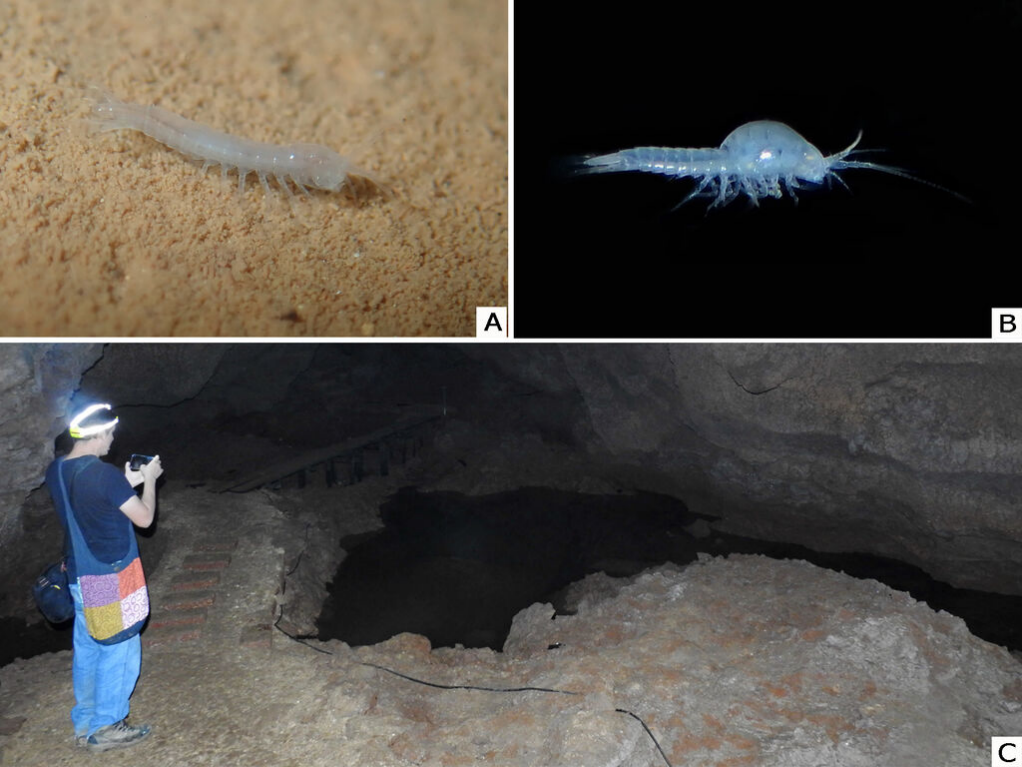
*Theosbaena loko* sp. n. A: Habitus in situ at Tham Loko. B: Lateral view of a female with brood pouch. C: General cave environment with the pools where the new species was collected.

Specimens were discovered in the dark zone of an isolated limestone of Tham Loko (Loko Cave), Khao Chiason District, Phatthalung Province (Fig. 1). This karst hill has been developed from Permian carbonate rock (286-245 mya) of the Ratchaburi group (Jantarit et al. 2020). The isolated limestone is relatively small, approximately 2.68 km long and 0.81 km wide (Fig. 2). The specimens were collected by hand and stored in 95% ethanol. Specimens were examined and dissected in 70% ethanol. All appendages were embedded in glycerine medium and mounted on a series of glass slides. Morphological characters were examined and drawn using a drawing tube attached to an Olympus CH30 light microscope. The pencil drawings were scanned and digitally inked using a WACOM Bamboo CTH-970 graphics board, following the method described in Coleman (2003). Photos of the habitus were taken by an Olympus Tough TG-5. Setae terminology follows Cals and Boutin (1985), Wagner (1994) and Rogers and Sanoamuang (2016).

Abbreviations used in the description are:

A- antenna; GN- gnathopod; MX- maxilla; MP- maxilliped, P- pereopod; PL- pleopod; T- telson and UR- uropod

**Repository**

NHM-PSU = Princess Maha Chakri Sirindhorn Natural History Museum, Prince of Songkla University, Songkhla, Thailand.

## Taxon treatments

### Theosbaena loko sp. n.

91813815-9781-553C-9F6D-94A6C2FAB706

144D7969-87BD-46A3-B3FF-A212230EB451

#### Materials

- **Type status:** Holotype. **Occurrence:** catalogNumber: PSUZC-PK6001-01-03; recordedBy: Koraon Wongkamhaeng; individualCount: 1; sex: male; lifeStage: adult; preparations: Specimen was examined and dissected in 70% ethanol. All appendages were embedded in glycerine medium and mounted on a series of glass slides.; **Taxon:** scientificName: Theosbaena loko; kingdom: Animalia; phylum: Arthropoda; class: Malacostraca; order: Thermosbaenacea; family: Halosbaenidae; genus: Theosbaena; specificEpithet: loko; **Location:** country: Thailand; stateProvince: Phatthalung; county: Thailand; locality: isolated limestone of Tham Loko (Loko Cave), Khao Chiason District, Phatthalung Province; verbatimElevation: 17 meters above sea level; locationRemarks: 25 Oct. 2017, dark zone of cave, by hand, leg. R. Promdam (sample # THA_SJ_PLG05); verbatimCoordinates: 7 26'53.2"N 100 07'30.5"E; georeferenceProtocol: label; **Identification:** identifiedBy: Koraon Wongkamhaeng; dateIdentified: 2020; **Event:** samplingProtocol: hand collecting; eventDate: 25/10/2017; **Record Level:** language: en; collectionCode: Crustaceans; basisOfRecord: PreservedSpecimen
- **Type status:** Paratype. **Occurrence:** catalogNumber: PSUZC-PK6001-04; recordedBy: Koraon Wongkamhaeng; individualCount: 1; sex: male; lifeStage: adult; preparations: Preserved in 70% ethyl alcohol; **Taxon:** scientificName: Theosbaena loko; kingdom: Animalia; phylum: Arthropoda; class: Malacostraca; order: Thermosbaenacea; family: Halosbaenidae; genus: Theosbaena; specificEpithet: loko; **Location:** country: Thailand; stateProvince: Phatthalung; county: Thailand; locality: isolated limestone of Tham Loko (Loko Cave), Khao Chiason District, Phatthalung Province; verbatimElevation: 17 meters above sea level; locationRemarks: 25 Oct. 2017, dark zone of cave, by hand, leg. R. Promdam (sample # THA_SJ_PLG05); verbatimCoordinates: 7 26'53.2"N 100 07'30.5"E; georeferenceProtocol: label; **Identification:** identifiedBy: Koraon Wongkamhaeng; dateIdentified: 2020; **Event:** samplingProtocol: hand collecting; eventDate: 25/10/2017; **Record Level:** language: en; collectionCode: Crustaceans; basisOfRecord: PreservedSpecimen
- **Type status:** Other material. **Occurrence:** recordedBy: Koraon Wongkamhaeng; individualCount: 6; sex: female; lifeStage: adult; preparations: Preserved in 70% ethyl alcohol; **Taxon:** scientificName: Theosbaena loko; kingdom: Animalia; phylum: Arthropoda; class: Malacostraca; order: Thermosbaenacea; family: Halosbaenidae; genus: Theosbaena; specificEpithet: loko; **Location:** country: Thailand; stateProvince: Phatthalung; county: Thailand; locality: isolated limestone of Tham Loko (Loko Cave), Khao Chiason District, Phatthalung Province; verbatimElevation: 17 meters above sea level; locationRemarks: 25 Oct. 2017, dark zone of cave, by hand, leg. R. Promdam (sample # THA_SJ_PLG05); verbatimCoordinates: 7 26'53.2"N 100 07'30.5"E; georeferenceProtocol: label; **Identification:** identifiedBy: Koraon Wongkamhaeng; dateIdentified: 2020; **Event:** samplingProtocol: hand collecting; eventDate: 25/10/2017; **Record Level:** language: en; collectionCode: Crustaceans; basisOfRecord: PreservedSpecimen

#### Description

**Male.** Body length 2.46 mm from head anterior margin to telson distal margin. Carapace reaching up to fourth pedigerous somite. Ocular scales present, broadly rounded, with longest dimension in medial third. Ocular scale 1.3x as long as broad, overlapping base of antenna I. Carapace extending to pereonite 2.

**Figure 3.**
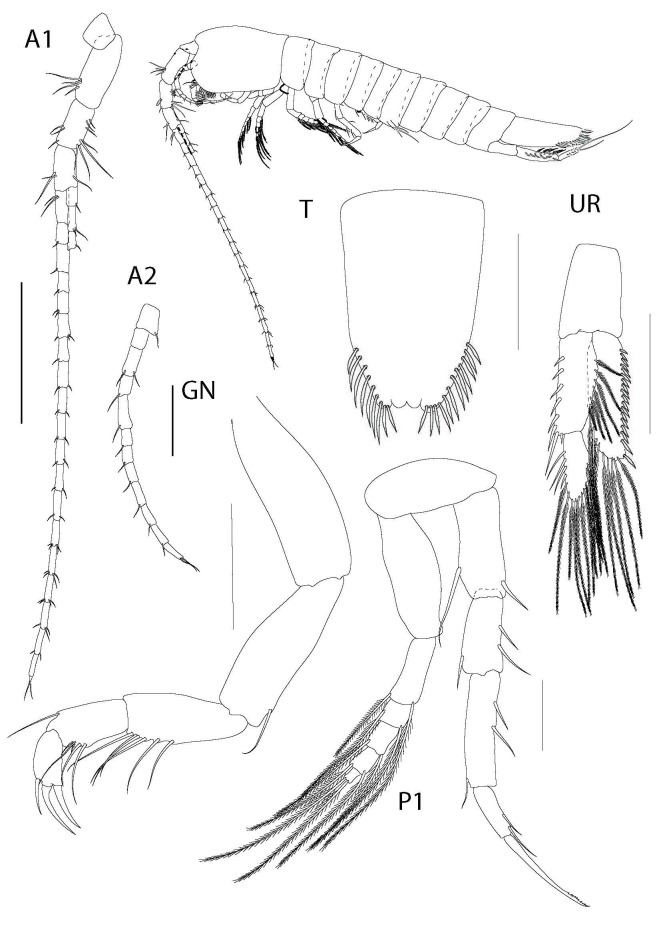
*Theosbaena loko* sp. n. 2.4 mm. A1, antenna 1; A2, antenna 2; GN, gnathopod; P1, Pereopod 2; UR, uropod; T, telson. All scale bars represent 0.2 mm.

Antenna 1 (Fig. 3) 1.2x body length, biramous, peduncle of 3 segments, lined with setae on dorsal and ventral sides. Primary flagellum with 20 segments, each with a distoapical seta; terminal segment on each flagellum with 2 apical setae, accessory flagellum with 12 segments.

**Antenna 2** (Fig. 3) uniramous, 0.3x body length, peduncle of 5 segments, distomedian margin of segment 1 with one simple seta (type IIA1). Flagellum with 7 segments, terminal segment with 2 apical setae.

**Labrum** round, 2.0x as long as broad, smooth, distal margin with fine, short microsetae. Labrum and labium without peculiarities.

**Labium** deeply cleft, margined with fine setae, with cleft margined with microsetae.

**Figure 4.**
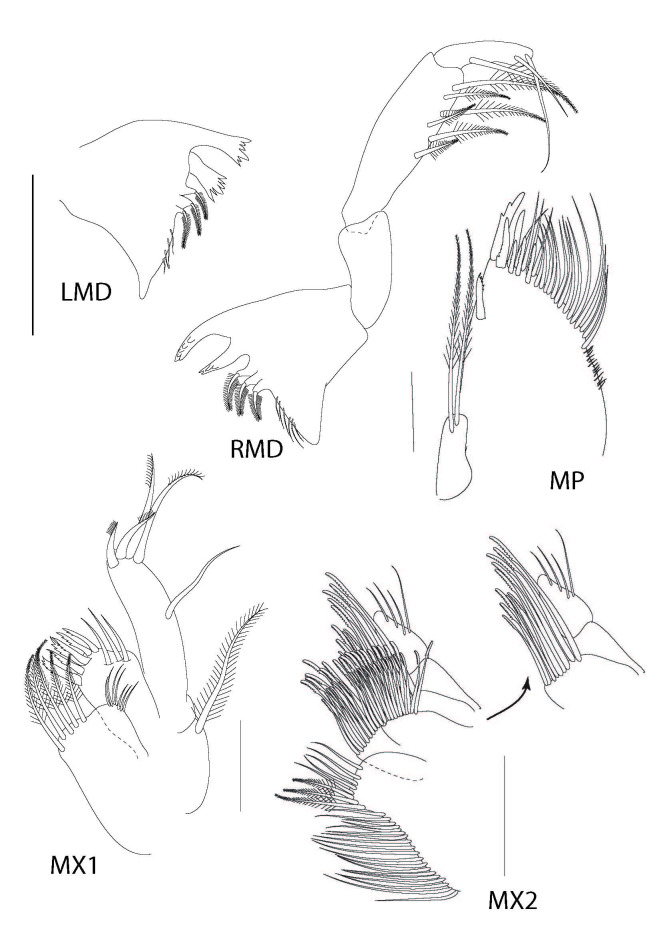
*Theosbaena loko* sp. n. 2.46 mm. LMD, left mandible; RMD, right mandible; MX1, maxilla 1; MX2, maxilla 2; MP, Maxilliped. All scale bars represent 0.2 mm.

**Mandible** (Fig. 4, LMD and 4RMD) with palp of 3 segments, ratio of segments 1–3 as 1:2:1; segments 1 subtriangular and unarmed; segment 2 elongate, medially extended, naked; segments 2 subcylindrical, lateral surface, distal portion bearing 6 macrosetae; segments 3 subcylindrical, subterminal bearing a plumose seta and distal bearing 2 plumose setae. Corpus mandibula left pars incisiva 6-dentate, right pars incisiva 5-dentate; left lacinia mobilis 4-dentate; right lacinia mobilis 6-dentate.

**Maxilla I** (Fig. 4, MX1) with coxal endite margined with a row of 5 plumose setae and 6 simple setae, respectively; basal endite with 2 rows of 6-toothed macrosetae and 3 simple setae;endopod unsegmented with one medial simple seta and apically lined with 4 plumose setae, 4x as long as proximal segment; exopod vestigial, represented by long plumose seta.

**Maxilla 2** (Fig. 4, MX2) coxal endite medial surface lined with 19 long plumose macrosetae, distal surface with 3 plumose setae and 5 simple setae; basipod with 2 endites, proximal endite bilobed, each apex margined with 2 rows of plumidenticulate macrosetae, basipodal endite 3 apex margined with longer plumidenticulate macrosetae. Endopodite of 2-segments; proximal segment inerm; distal segment with 4 simple setae.

**Maxilliped** (Fig. 4, MP) exopod reduced, longer than broad, with 2 elongate pectinate macrosetae. Endopod vestigial, represented by isolated seta. Basal endite broad, lateral margin straight, apical fifth of anterior surface and distal margin with stout pectinate spines and macrosetae; more macrosetae medially than at apex, apex with more spines.

**Gnathopod** (Fig. 3, GN) uniramous, basoischium elongate, 4Í as long as broad, innerm. Merus subequal to basoischium. Carpus expanded distally, bearing a longitudinal row of 5 simple setae macrosetae on medial margins; each seta shorter than carpus. Propodus suboval, bearing longitudinal row of 5 simple setae on medial margin; each seta subequal to the length of propodus. Dactylus suboval, distal margin convex. Unguis formed by 3 curved spines at distolateral corner. Dactylus distomedial angle inerm.

**Pereopod II** (Fig. 3) coxa not pronounced, rounded. Exopod 0.8x length of endopod, composed of 2 segments: basal segment suboval, inerm. Segment 4-6 distal corner with plumose macrosetae. Basis subcylindrical, with one medial seta and one distal seta, 3Í as long as wide. Ischiomerus rectangular, twice as long as width, bearing one medial seta and one distal seta. Carpus subcylindrical, not expanded distally, 1.7x longer than width, bearing 2 medial and one lateral distal spiniform macrosetae. Propodus anterioposteriorly flattened, approximately 4x longer than width, medial margin bearing 2 setae and distal corner with one seta. Propodus lateral margin bearing 2 subapical macrosetae distally, each about as long as segment’s width. Dactylus anterioposteriorly flattened, margins converging into truncated apex; apex half as wide as base. Dactylus apex with single elongate spine (unguis), twice as long as dactylus.

**Figure 5.**
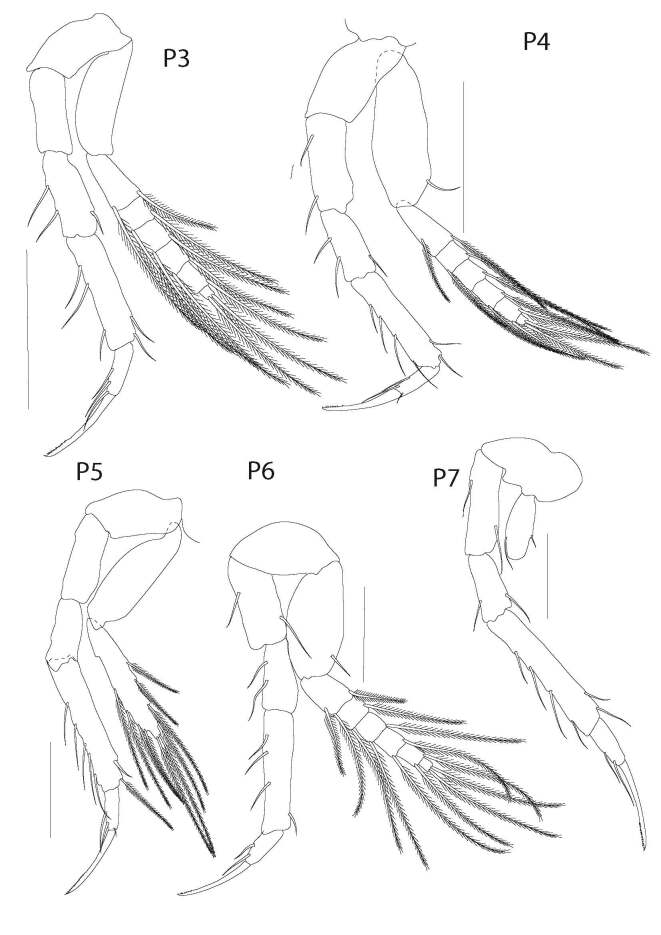
*Theosbaena loko* sp. n. 2.46 mm. P3, pereopod 3; P4, pereopod 4; P5, pereopod 5; P6, pereopod 6; P7, pereopod 7. All scale bars represent 0.2 mm.

**Pereopods III** (Fig. 5) through IV similar to that of pereopod II. Pereopod V coxa not pronounced, rounded. Exopod 0.8x length of endopod, composed of 2 segments; proximal segment subrectangular, not expanded medially, inerm. Distal segment lanceolate, submargined in long, plumose macrosetae. Basis subcylindrical, inerm, twice as long as width. Ischiomerus subcylindrical, 3x as long as width, inerm. Carpus subcylindrical, expanded distally, 1.5x longer than width, inerm. Propodus anterioposteriorly flattened, approximately 6x longer than width, medial margin bearing a longitudinal row of well-spaced, spiniform macroseta, each subequal to segment’s width. Propodus lateral margin bearing 2 subapical macrosetae, each longer than segment’s width. Dactylus anterioposteriorly flattened, margins converging to a truncated apex; apex 0.7x as wide as base. Dactylus apex serrate, innerm.

**Pereopod VI** (Fig. 5) coxa not pronounced, rounded. Exopod 0.8x length of endopod, composed of 7 segments: proximal segment suboval, medially expanded, with single macroseta at distolateral corner. Segment 2-7 subrectangular, distal corner with plumose macrosetae. Basis suboval, inerm, twice as long as width. Ischiomerus subcylindrical, 2.3x as long as width, bearing 2 medial setae. Carpus subcylindrical, not expanded distally, 1.4x longer than width, bearing 2 macrosetae medially. Propodus anterioposteriorly flattened, approximately 6x longer than width, medial margin bearing a longitudinal row of spiniform macroseta, each subequal to segment’s width. Propodus lateral margin bearing 2 subapical macrosetae, each about twice as long as segment’s width. Dactylus anterioposteriorly flattened, margins converging to a truncated apex; apex 0.7x as wide as base. Dactylus apex serrated, innerm.

**Uropod** (Fig. 3) Endopod ovate, one articulate, longer unsegmented than protopod; lateral margin bearing 18 cuspidate setae; distomedial margin bearing long, plumose macrosetae, equal in length to endopod; distolateral margin bearing seven elongate plumose macrosetae. Exopod 2-segmented, first segment longer than second segment; proximal segment with straight lateral margin armed with row of stout spines; medial margin convex, widest at middle, with row of plumose macrosetae; Distal segment subovate, lateral margined with elongate, plumose macrosetae; medial edge margined with stout spines, 0.8x length of segment.

**Telson** (Fig. 3) longer than broad, 1.8x longer than basal broad, tapering, distally, terminal concave, anal lobes protrude beyond the terminal stretch. Left and right subterminal margin with 9 and 12 cuspidate setae.

#### Diagnosis

*Theosbaena loko* sp. n. is the third species of the genus reported from Thailand. *Theosbaena loko* sp. n. can be distinguished from its congeners in having a telson 1.8x longer than its breadth, maxilla 1 palp distal segment 4x as long as proximal palpomere and a maxillopodal exopod twice as long as its basal width. It shares some characteristics with *T. cambodjiana* in having: mandibular palp segment 1:2:1 ratio 9:1.5:7/ segment 2 with 6 plumose setae; ocular scale evenly arcuate and rounded; gnathopod dactylus subrectangular, with 3 long, stout, arcuate macrosetae, each bearing a ventral membrane and uropod distal segment of the exopod and the distal margin of the endopod both bearing elongate, plumose macrosetae and endopod lateral edge is margined with a row of scaliform macrosetae. However, *T. loko* sp. n. differs from *T. cambodjiana* in the absence of pleopod 1. *Theosbaena loko* sp. n. is similar to *T. kiatwongchai* in having: a mandibular palp segment 1:2:1 ratio 9:1.5:7/ segment 2 with 6 plumose setae (vs. palp segment 1:2:3 ratio 9:1.5:7/ segment 2 with 6 microsetae); pereopod 1-4 exopod contains more than 2 segments (vs. 2 segments); ocular scale evenly arcuate and rounded (vs. transverse); and uropod endopod lateral edge is margined with a row of scaliform macrosetae (vs. endopod medial margin is inerm, except for 2 filiform macrosetae, midway along its length). Diagnostic morphological characters and their variation for each population/species are given in Table 1 and the identification key of the genus *Theosbaena* is provided.

#### Distribution

*Theosbaena loko* sp. n. is only known from the freshwater pool in the dark zone of Loko Cave, Khao Chaison District, Phatthalung Province. The Cave is 352 metres long. The Cave contains three pools, with *T. loko* sp. n. found in all three pools, although two of the pools dry out during the dry season.

#### Ecology

The new species was found swimming and walking on the clay substrate of the pool in the dark zone of the Cave. The physical factors in the pool were as follows: Temperature (25.1–25.7^0^C); conductivity (217–282 µS); total dissolved solids (146–182 ppm); salinity (108–137 ppm); dissolved oxygen (6.0–8.2 mgO_2_/l); pH (7.98–8.22); turbidity (8–12 FAU); water hardness (99–150 mg/l CaCO_3_); and CaCO_3_ (70.20–85.40 mg/l). The new species co-occurs with stygobiotic isopod *Stenasellus* sp., three species of Rotifer: *Lecane bulla* (Gosse, 1851); *Lecane hamata* (Stokes, 1896); *Lecane quadridentate* (Ehrenberg, 1830), a species of Daphniidae (*Scapholeberis kingi* Sars, 1888) and undetermined Cyclopidae. Moreover, two fish species were observed: *Barbodes binotatus* (Valenciennes, 1842) and *Rasbora paviana* Tirant, 1885. These fish may be potential predators of this microshrimp. The co-occurrence of stygobiotic fauna in the same area is not exceptional and *T. cambodjiana* was also reported to live in the same pool with the isopod *Stenasellus cambodianus* Boutin & Magniez, 1985. Additionally, *T. cambodjiana* in Khon Kaen, Thailand occurs with five other stygobiotic species in the same pond, i.e. *Dugesia deharvengi* Kawakatsu & Mitchell, 1989, *Heterochaetella glandularis* (Yamaguchi, 1953), *Aequigidiella aquilifera* Botosaneanu & Stock, 1989, *Stenasellus rigali* Magniez, 1991 and *Siamoporus deharvengi* Spangler, 1996 (Deharveng and Bedos 2012). Unfortunately, there are no ecological data for *T. kiatwongchai.* The authors attempted to access the habitat of *T. kiatwongchai* in August 2020, but unfortunately, the cave access was dangerous and the Forest Park staff claimed that the air was unsuitable, meaning that it was impossible to undertake a fauna and habitat evaluation of the cave.

#### Conservation

The researchers herein propose *T. loko* sp. n. as an endangered species according to the IUCN Standards and Petitions Committee (2019) criteria. This status is proposed because its population size is small (only seven captured specimens from five observations). The new species is highly endemic to the permanent pool in the Loko Cave and the discovery considerably extends the narrow geographic occurrence of the genus (Fig. 1). The karst hill and the Cave are surrounded by agricultural areas, such as paddy fields and rubber and orchard plantations, where agricultural practices and anthropogenic activities have significantly increased. Today the Cave has become a tourist attraction where lights and simple infrastructure inside the Cave have been introduced. The habitat is, therefore, threatened in the face of growing anthropogenic disturbance. Interestingly, this Cave has one of the richest fauna in Thailand, harbouring at least 79 species of cave fauna with many species that are unknown to science (Jantarit et al. 2020). The Cave also hosts Pendlebury's roundleaf bat, *Hipposideros pendleburyi* Chasen, 1936, which has been assessed as a vulnerable species by the IUCN. Hence, the description of this new species not only emphasises the high level of endemism in this cave, but also has implications for environmental awareness, developing policy for cave conservation strategies, together with promoting ecotourism in the areas.

## Identification Keys

### Key to species of the genus *Theosbaena*

**Table:** 

1	Uropod endopod with elongate plumose macrosetae shorter than exopod, lateral edge margined with small spiniform macrosetae and two filiform plumose setae at mid-length	*T. kiatwongchai*
–	Uropod endopod with elongate plumose macrosetae subequal to exopod, lateral edge is margined with a row of scaliform macrosetae	2
2	Telson entire	*T. cambodjiana*
–	Telson apically emarginate	*T. loko*

## Supplementary Material

XML Treatment for Theosbaena loko

## Figures and Tables

**Table 1.** Diagnostic morphological characters of known *Theosbaena* species and their variation for each population/species (N/A indicates no information).

**Characters/species**	***Theosbaena cambodjiana***	***Theosbaena kiatwongchai***	***Theosbaena loko* sp. n.**
Flagellum on antenna 1	29	25	19
Accessory flagellum	14	13	10
Ocular scales	evenly arcuate and rounded	more transverse, with the widest point being just medial to the centre-line of the structure.	evenly arcuate and rounded
**Mandible**			
Palp segment	1:2:1	1:2:3	1:2:1
Setae	8 plumidenticulate macrosetae	6 microsetae	6 plumose macrosetae
Corpus mandibula	L- 6 dentate, R-4 dentate	N/A	L- 6 dentate, R-5 dentate
**Maxilla I** palp- distal palpomere: proximal palpomere	subequal	twice	four times
Maxillopodal exopod	1.5 times its basal width	greatly reduced, as long as width and the point of articulation is not clear; fused	twice as long as basal width
**Gnathopod dactylus**			
shape	subrectangular	subtriangular, widest apically	subrectangular
membranous macrosetae	present (3)	absent	present (3)
dorsoapical ungual spine	absent	present	present
ventroapical setal tuft	absent	present	present
distal margin	widened and somewhat flattened	convex	convex
distomedial angle	N/A	a row of short setae	inerm
unguis	three arced (modified) serrulate macrosetae (type IIB1) with a prominent membrane ventrally.	single curved spine at the distolateral corner.	three curved spines at the distolateral corner.
**Pereopod 7**			
coxa	not pronounced	pronounced	not pronounced
Exopod: endopod	N/A	0.8	0.8
Basal segment	N/A	rectangular, with a spine at distolateral corner	suboval, inerm.
Penial lobe	subcylindrical and slightly bent	straight and fusiform	simple, naked, tall, almost straight
**Ple I**	present	absent	absent
**Uropod**			
Endopod	Oval, distal segment bearing elongate plumose macrosetae subequal to endopod, lateral edge is margined with a row of scaliform macrosetae.	Oval, distal segment bearing elongate plumose macrosetae shorter than exopod, lateral edge margined with small spiniform macrosetae and two filiform plumose setae at mid-length.	Suboval, distal segment bearing elongate plumose macrosetae subequal to exopod, lateral edge is margined with a row of scaliform macrosetae.
Exdopod	Distal segment subovate, lateral margin with elongate plumose macrosetae, medial edge without spines; segment apex with single elongate spine 0.8x the length of the segment.	Distal segment subovate, lateral margin with elongate, plumose macrosetae; medial edge margined with spines; segment apex with single elongate spine 0.8x length of segment.	Distal segment subovate, lateral margin with elongate, plumose macrosetae; medial edge margined with stout spines; segment apex without elongate spine.
**Telson**	entire	emarginate	emarginate
Telson length/width ratio	0.8	1.2	1.8
Telson apex	apical region margined with macrosetae, apex entire.	apex lobes have curved, rigid spines that arc medially towards the medial cleft	apex lobes have curved, rigid spines that arc medially towards the medial cleft
